# Short-term outcome after cystectomy: comparison of early oral feeding in an enhanced recovery protocol and feeding using Bengmark nasojejunal tube

**DOI:** 10.1007/s00345-017-2133-2

**Published:** 2017-11-22

**Authors:** C. S. Voskuilen, E. E. Fransen van de Putte, J. Bloos-van der Hulst, E. van Werkhoven, W. M. de Blok, B. W. G. van Rhijn, S. Horenblas, R. P. Meijer

**Affiliations:** 1 Department of Urologic Oncology, University Medical Center Utrecht Cancer Center, Utrecht, The Netherlands; 2 Department of Urology, The Netherlands Cancer Institute—Antoni van Leeuwenhoek Hospital, Amsterdam, The Netherlands; 3 Department of Biometrics, The Netherlands Cancer Institute—Antoni van Leeuwenhoek Hospital, Amsterdam, The Netherlands

**Keywords:** Bladder cancer, Perioperative care, Enteral feeding, Postoperative complications, Enhanced recovery after surgery

## Abstract

**Purpose:**

Cystectomy for bladder cancer is associated with a high risk of postoperative complications. Standardized perioperative protocols, such as enhanced recovery after surgery (ERAS) protocols, aim to improve postoperative outcome. Postoperative feeding strategies are an important part of these protocols. In this two-centre study, we compared complications and length of hospital stay (LOS) between an ERAS protocol with early oral nutrition and a protocol with early enteral feeding with a Bengmark nasojejunal tube.

**Methods:**

We retrospectively reviewed 154 consecutive patients who underwent cystectomy for bladder cancer in two hospitals (Hospital A and B) between 2014 and 2016. Hospital A uses an ERAS protocol (*n* = 45), which encourages early introduction of an oral diet. Hospital B uses a fast-track protocol comprising feeding with a Bengmark nasojejunal tube (Bengmark-protocol, *n* = 109). LOS and complications according to Clavien classification were compared between protocols.

**Results:**

Overall 30-day complication rates in the ERAS and Bengmark protocol were similar (64.4 and 67.0%, respectively; *p* = 0.463). The rate of postoperative ileus (POI) was significantly lower in the Bengmark protocol (11.9% vs. 34.4% in the ERAS protocol, *p* = 0.009). This association remained significant after adjustment for other variables (odds ratio 0.32, 95% confidence interval 0.11–0.96; *p* = 0.042). Median LOS did not differ significantly between protocols (10 days vs. 11 days in the ERAS and Bengmark protocols, respectively; *p* = 0.861).

**Conclusions:**

Early oral nutrition in Hospital A was well tolerated. However, the Bengmark protocol was superior with respect to occurrence of POI. A prospective study may clarify whether the lower rate of POI was due to the use of early nasojejunal tube feeding or other reasons.

## Introduction

Radical cystectomy (RC) for bladder cancer (BC) is associated with a high complication rate. Thirty-day overall complication rates vary from 26 to 78% with mortality rates of 1.0–4.0% [1–3]. The most common complications are infectious or gastrointestinal related, with postoperative ileus (POI) as one of the most frequent [4]. POI is an important reason for prolonged length of hospital stay (LOS) after RC [4–6].

In recent years, attempts have been made to improve recovery and reduce LOS by introducing enhanced recovery after surgery (ERAS) programmes. Their objective is to minimize physiologic stress effects in major surgery and thereby decrease time to return of normal function. Postoperative feeding strategies are an important part of these protocols and usually comprise oral intake within 24 h after surgery. In clinical practice, however, perioperative intake differs greatly between ERAS protocols [7, 8].

In the current study, perioperative protocols for RC in two hospitals were compared. Hospital A is an academic hospital with an annual number of 25 cystectomies for BC. Hospital B is a tertiary national referral cancer hospital. In this hospital, over 60 cystectomies for BC are performed annually. There is a close collaboration between the oncologic urology departments of both hospitals in a multidisciplinary tumor board and in research but their perioperative protocols for RC differ. In Hospital A, the traditional perioperative protocol was replaced by an ERAS protocol in 2014. In this ERAS protocol, oral diet is started the day after surgery, when tolerated by the patient. Hospital B uses a protocol comprising early enteral feeding via a Bengmark nasojejunal tube (Bengmark protocol). The aim of this study was to compare postoperative outcomes and LOS of RC patients in an ERAS protocol comprising early oral nutrition and in a protocol comprising early enteral feeding via a Bengmark tube.

## Methods

All consecutive patients who underwent cystectomy for BC between January 2014 and October 2016 in Hospital A or B were included. Both open and robot-assisted procedures were analysed. Patients who needed an adjunctive procedure (e.g., nephroureterectomy) were excluded. In addition, patients who received an ureterocutaneostomy were excluded, as this procedure does not include bowel surgery. Comorbidity was assessed by the Charlson Comorbidity Index (CCI) [9]. Hospital stay was measured from the day of admission until discharge after RC. Differences in perioperative care between the protocols are highlighted below. An overview of all elements of both protocols is provided in the Appendix.

### Preoperative care

In the ERAS protocol (Hospital A), patients were admitted the morning of surgery. They did not receive bowel preparation. All patients, excluding insulin-dependent diabetics, were administered 400 cc of a carbohydrate rich drink 2–3 h prior to surgery.

In Hospital B, patients were admitted 1 day before surgery to place a nasojejunal feeding tube (Bengmark). This is a self-propelling, auto-positioning post-pyloric feeding tube. In addition, patients in this hospital were treated with selective digestive decontamination (SDD; see Appendix).

### Surgery

Surgical teams of both hospitals were equally trained and experienced. In both hospitals, surgeons adhered to the same surgical techniques for both open radical cystectomy (ORC) and robot-assisted radical cystectomy (RARC) including similar pelvic lymph node dissection templates. In hospital A, patients were operated by one of two staff urologists. In hospital B, patients were operated by one of four staff urologists. In this cohort, RARC was performed by one urologist in hospital A and two urologists in hospital B.

Both protocols used a combination of general and regional anaesthesia, with insertion of a thoracic epidural for postoperative pain management. The ERAS protocol underwent revision regarding epidural analgesia in November 2015, omitting a thoracic epidural in patients undergoing a robot-assisted procedure. Blood loss and operation time were measured.

### Postoperative care

In the ERAS protocol, the nasogastric tube (NGT) was removed directly after surgery. Patients were allowed to try a normal diet on POD 2. Until normal intake was achieved, they were advised to take 1–2 high calorie nutritional drinks (Nutridrink^®^). To prevent POI, patients were given chewing gum three times a day and magnesium oxide two times a day. Epidural analgesia, if administered, was replaced by non-opioid pain control 48 h after surgery. Early mobilisation was promoted, aiming for two hours out of bed on POD 1 and at least 6 h out of bed from POD 2 onwards.

In the Bengmark protocol, the NGT was removed within 24 h after surgery. Patients started with enteral nutrition at 20 ml/h via the Bengmark tube on POD 1. On POD 2, enteral nutrition was raised to 40 ml/h and patients were encouraged to eat soft foods. From day 3 to day 5, enteral nutrition was gradually raised to 60 ml/h and patients were allowed to eat normally if possible. Enteral nutrition was stopped if normal intake was achieved. Epidural analgesia was stopped on POD 4. Duration of enteral nutrition via the Bengmark tube was recorded. In both protocols, time to removal of NGT and time to last drain removal were recorded. Finally, in both hospitals, patients were discharged if they had met predefined criteria (Appendix).

### Complications

Hospital and outpatient clinical records were reviewed in detail and complications and unplanned readmissions occurring within 30 and 90 days of surgery were recorded. All complications were graded according to the Clavien–Dindo grading system [10]. POI was defined as requirement for cessation of an oral intake regime for > 24 h, the need for a NGT and/or absence of bowel function beyond POD4.

### Statistical analysis

Statistical analyses were performed using IBM SPSS Statistics version 21.0. (Armonk, NY, IBM Corp.). Normally and non-normally distributed data were analysed using independent *t* tests and Mann–Whitney *U* tests, respectively. Categorical data were analysed using Chi-squared tests. Associations of protocol and surgical factors with the occurrence of complications were determined using univariable logistic regression. Multivariate logistic regression was used to identify independent effect of protocol on POI. Co-variates included age, ASA scores, surgical approach and use of epidural analgesia. Statistical significance was defined as a *p* value < 0.05.

## Results

### Patient characteristics

In Hospital A, 50 patients were treated in the ERAS protocol versus 121 patients in Hospital B in the Bengmark protocol. Forty-five patients in the ERAS protocol and 109 patients in the Bengmark protocol met our predefined inclusion criteria. Patient characteristics are shown in Table 1. Patients in the ERAS protocol were significantly older than patients in the Bengmark protocol (mean 69.9 and 64.9 years, respectively; *p* = 0.005) and had higher ASA scores (ASA 3: 28.9 and 11.0%, respectively; *p* = 0.001). There were significantly more patients in the Bengmark protocol who received neoadjuvant chemotherapy (49.5% vs. 24.4% in the ERAS protocol, *p* = 0.007). Furthermore, in the Bengmark group, more patients were previously exposed to pelvic radiation (23.9% vs. 8.6% ERAS-protocol, *p* = 0.043).

**Table 1:** Patient characteristics

	ERAS (*n* = 45)	Bengmark (*n* = 109)	*p* value
Age, years			
Mean (± SD)	69.9 (10.0)	64.9 (9.9)	0.005
BMI, kg/m^2^			
Mean (± SD)	25.4 (4.8)	26.0 (3.7)	0.421
	*n* (%)	*n* (%)	
Sex			
Male	34 (75.6)	79 (72.5)	0.847
Female	11 (24.4)	30 (27.5)	
Diabetes			
Yes	7 (15.6)	9 (8.3)	0.289
No	38 (84.4)	100 (91.7)	
Charlson Comorbidity Index			
0	21 (46.7)	70 (64.2)	0.117
1	14 (31.1)	25 (22.9)	
≥ 2	10 (22.2)	14 (12.8)	
Diversion type			
Bricker	44 (97.8)	86 (78.9)	0.009
Neobladder	1 (2.2)	17 (15.6)	
Indiana pouch	0	6 (5.5)	
T-stage before surgery			
≤ T2	28 (62.2)	61 (56.0)	0.592
≥ T3	17 (37.8)	48 (44.0)	
N-stage before surgery			
Negative	38 (84.4)	94 (86.2)	0.988
Positive	7 (15.6)	15 (13.8)	
Neoadjuvant chemotherapy			
Yes	11 (24.4)	54 (49.5)	0.007
No	34 (75.6)	55 (50.5)	
Previous pelvic radiation*			
Yes	4 (8.6)	26 (23.9)	0.043
No	41 (91.4)	83 (76.1)	
ASA score			
ASA 1	3 (6.7)	33 (30.3)	0.001
ASA 2	29 (64.4)	64 (58.7)	
ASA 3	13 (28.9)	12 (11.0)	

*ASA* American Society of Anesthesiologists, *BMI* Body mass index, *SD* standard deviation

*Salvage cystectomies after radiotherapy included

### Surgical and postoperative details

In Table 2, surgical and postoperative details are shown. A robotic approach was used approximately twice as often in the ERAS protocol (64.4% vs. 27.5% in the Bengmark protocol; *p* < 0.001). Epidural analgesia was used less frequently in the ERAS protocol (64.4% vs. 99.1%; *p* < 0.001). Median LOS did not differ significantly (10 days vs. 11 days for ERAS vs. Bengmark, respectively; *p* = 0.861). Comparing RARC and ORC within the protocols, LOS was shorter in RARC in both the ERAS and Bengmark protocols. This difference between open and robotic surgery was most apparent in the ERAS protocol (9 days vs. 15.5 days for RARC and ORC in the ERAS group, respectively; 10 days vs. 11 days for RARC and ORC in the Bengmark group, respectively).

**Table 2:** Surgical and postoperative details

	ERAS	Bengmark	*p*-value
Robot-assisted approach, *n* (%)			
No	16 (35.6)	79 (72.5)	< 0.001
Yes	29 (64.4)	30 (27.5)	
Epidural analgesia, *n* (%)			
Overall	29 (64.4)	108 (99.1)	< 0.001
ORC	14 (87.5)	79 (100)	0.027
RARC	15 (51.7)	29 (96.7)	< 0.001
Median duration of surgery, min (range)			
Overall	340 (180–510)	243 (145–480)	< 0.001
ORC	240 (180–380)	240 (145–480)	0.494
RARC	360 (285–510)	285 (180–450)	0.001
Median blood loss, cm^3^ (range)			
Overall	400 (50–2000)	800 (10–4900)	0.010
ORC	850 (400–2000)	1100 (50–4900)	0.221
RARC	200 (50–900)	125 (10–1100)	0.016
Median LOS, days (range)			
Overall	10 (8–79)	11 (8–52)	0.861
ORC	15.5 (8–52)	11 (8–52)	0.183
RARC	9 (8–79)	10 (8–22)	0.752
NGT removal, POD, median (range)	0 (0–8)	1 (0–15)	< 0.001
Patients requiring NGT replacement, *n* (%)	14 (31.1)	20 (18.3)	0.128
Epidural removal, POD, median (range)	2 (1–5)	5 (2–11)	< 0.001
Patients with enteral tube feeding, *n* (%)	5 (11.4)	103 (94.5)	< 0.001
Duration of enteral tube feeding, days, median (range)	5 (4–6)	5 (0–26)	0.651
Patients with TPN, *n* (%)	12 (26.7)	14 (13.0)	0.069
Duration of TPN, days, median (range)	10 (6–28)	7 (2–25)	0.039

*LOS* length of hospital stay, *NGT* nasogastric tube, *ORC* open radical cystectomy, *POD* postoperative day, *RARC* robot-assisted radical cystectomy, *TPN* total parenteral nutrition

In the ERAS protocol, the NGT was removed right after surgery in most cases, but 30.4% of patients required replacement of the NGT. More patients in the ERAS protocol needed total parenteral nutrition (TPN), although the difference was not statistically significant (26.7% vs. 13.0% in the Bengmark protocol; *p* = 0.069). Median duration of epidural analgesia was 2 days in the ERAS protocol (*n* = 29) compared to 5 days in the Bengmark protocol (*n* = 103) (*p* < 0.001.)

### Complications and readmissions

In Table 3, complications occurring within 30 days after RC are shown according to protocol, with multiple complications in some patients. Furthermore, 30-day- and 90-day readmission rates are shown. Overall complication rates were similar (64.4% vs. 67.0% for the ERAS and Bengmark protocols, respectively; *p* = 0.763). The percentage of POI was significantly lower in the Bengmark protocol (11.9% vs. 31.4% in the ERAS protocol; *p* = 0.009). In univariable logistic analysis, only the Bengmark protocol was significantly associated with a lower risk of POI (odds ratio (OR) 0.30, 95% confidence interval (CI) 0.13–0.71; *p* = 0.006). This association remained significant after adjusting for age, surgical approach, higher ASA scores and the use of epidural analgesia (OR 0.32, 95% CI 0.11–0.96; *p* = 0.042, Table 4). Readmission rates were higher in the Bengmark group, although the differences were not statistically significant (*p* = 0.136). Urinary tract infection was the most common reason for readmission in both hospitals (data not shown).

**Table 3:** Complications, return to theatre and readmissions

	ERAS *n* (%)	Bengmark *n* (%)	*p*-value
Overall complication rate ≤ 30 days	29 (64.4)	73 (67.0)	0.763
Return to theatre ≤ 30 days	8 (17.8)	11 (10.1)	0.187
Minor complications ≤ 30 days^a^			
Ileus	14 (31.4)	13 (11.9)	0.009
Urinary tract infection	5 (11.1)	19 (17.4)	0.325
Wound infection	3 (6.7)	4 (3.7)	0.417
Blood transfusion	4 (8.9)	20 (18.3)	0.141
Pneumonia	6 (13.3)	6 (5.5)	0.110
Atrial fibrillation	2 (4.4)	3 (2.8)	0.630
Delirium	2 (4.4)	6 (5.5)	1
Major complications ≤ 30 days^a^			
Intestinal suture leakage	3 (6.7)	2 (1.8)	0.124
Fascial dehiscence	4 (8.9)	3 (2.8)	0.216
Ureteroileal leakage requiring drainage	5 (11.1)	15 (13.8)	0.906
Lymphocele requiring drainage	3 (6.7)	5 (4.6)	0.253
Pelvic/abdominal abscess	0	1 (0.9)	1
Bleeding	0	4 (3.7)	0.322
Sepsis	0	6 (5.5)	0.181
Pulmonary embolus	1 (2.2)	0	0.292
Renal failure	0	2 (1.8)	1
Cerebrovascular accident	0	1 (0.9)	1
Clavien grade ≤ 30 days^b^			
No complications	16 (35.6)	36 (33.0)	0.767
I–II	18 (40.0)	40 (36.7)	
≥ III	11 (24.4)	33 (30.3)	
Clavien grade 31–90 days^b^			
No complications	40 (88.9)	91 (84.3)	0.868
I–II	3 (6.7)	9 (8.3)	
≥ III	2 (4.4)	8 (7.4)	
Readmissions			
Within 30 days	3 (6.7)	17 (15.6)	0.134
Within 90 days	8 (17.8)	32 (29.4)	0.136

^a^Some patients experienced multiple complications

^b^If more than one complication occurred in one patient, the highest grade was scored

**Table 4:** Multivariable logistic regression analysis identifying factors associated with postoperative ileus

	OR	95% CI	*p* value
Bengmark protocol	0.32	0.11–0.96	0.042
Robot-assisted approach	0.70	0.24–2.00	0.500
ASA II	0.80	0.25–2.58	0.710
ASA III	0.86	0.20–3.76	0.840
Epidural analgesia	0.85	0.21–3.45	0.820
Age (increase of 1 year)	1.05	1.00–1.10	0.050

## Discussion

Enhanced recovery protocols after RC are widely used and have led to improved overall complication rates and shorter LOS [11]. However, for some individual ERAS components, such as postoperative feeding strategies, evidence from the literature is sparse. In this retrospective study, we compared complications and LOS between an ERAS protocol with early oral nutrition and a protocol with early enteral feeding with a Bengmark nasojejunal tube. The latter was superior with respect to occurrence of POI, while overall complication rates and LOS were similar.

Overall complication rates in our study were in the higher range of earlier reported rates, which vary between 26 and 78% [1–3]. However, definition and types of reported complications differ between studies and are subject to the thoroughness of registration. Evaluating a specific complication such as POI, our rates are also in the higher range of previous series, specifically considering the POI rate of 31.4% in the ERAS protocol. In a systematic review, POI incidence after RC ranged from 1.6 to 23.5% [4]. However, the definition of POI is highly variable across urologic literature, and therefore, true incidence is hard to determine [12]. In our study, clinical records were reviewed in detail and POI was scored if our strict predefined criteria (see Methods) were met. Nevertheless, when studying POI retrospectively, observation bias cannot be excluded. The higher POI rate in the ERAS protocol may partly be explained by the fact that introduction of a new perioperative protocol (i.e., ERAS) makes caregivers more conscious of complications and LOS. Several previous reports on ERAS protocols have demonstrated this effect [13, 14].

Notwithstanding this limitation, it is interesting that we found a lower rate of POI in the Bengmark group. Most ERAS protocols in urologic surgery are adapted from protocols in colorectal surgery. In this field, many high-quality clinical studies have shown that early oral intake as a route for enteral nutrition is safe and effective [15]. However, these data may not be directly applicable to RC because the construction of a urinary diversion, the uretro-enteric anastomosis, potential urinary leakage and large pelvic dissection differ between RC and colorectal surgery. Early introduction of enteral feeding is inherent to any enhanced recovery protocol because of positive effects on insulin resistance, muscle function and wound healing; the latter being specifically relevant to the integrity of the bowel anastomosis after creation of a urinary diversion [15–17]. Now the question is, which route of enteral nutrition should be preferred. Only one study prospectively reviewed the impact of early oral feeding on complications and LOS after RC [18]. In this randomized trial, patients either received access to liquids and then a regular diet on POD 1 and further (*n* = 50), which is comparable to the ERAS protocol in the current study, or care as usual with introduction of a liquid diet after return of bowel activity (*n* = 52). Although the trial did not meet the enrolment target, no differences in complications (including POI) were found and early oral feeding was well tolerated [18].

Apart from our study, no other studies have evaluated the outcome of early enteral tube feeding in RC patients. Nasojejunal early nutrition was introduced in Hospital B after a meta-analysis of studies in abdominal surgery showed decreased mortality in patients who were fed enterally compared to patients without enteral feeding [19, 20]. In the literature, no causal relation of nasojejunal enteral feeding and lower ileus rates has been described. We hypothesize that after creation of a urinary diversion, a period of gastroparesis may develop, which may be circumvented by the nasojejunal enteral feeding. Whereas, a lower POI rate may be interpreted as an advantage of the Bengmark protocol, there are downsides to consider. First, despite the fact that the Bengmark tube is an auto-positioning device in the presence of normal gastric motility, it remains an invasive procedure with possible complications. Second, the tube has to be inserted at least 12 h prior to surgery, because of the time the self-propelling mechanism takes to migrate into the jejunum. Consequently, patients need to be admitted the day before surgery. Finally, feeding tubes cause nasopharyngeal discomfort in the postoperative course.

In our study, the association between the Bengmark protocol and the lower rate of POI was independent of other factors, such as epidural use. Previous studies in colorectal surgery have suggested that postoperative epidural analgesia, in contrast to opiate use, can lead to a decrease in ileus [21]. Further research should be undertaken to investigate the effects of different pain medications on RC patients.

Main limitations of this study are its retrospective character, the limited sample size in one of the arms and the differences in patient and surgical characteristics between the two centers. Since Hospital B is a comprehensive cancer center, more patients in this hospital underwent neoadjuvant chemotherapy or had a history of pelvic radiation. Another difference is due to the surgical approach, with more patients undergoing RARC in the ERAS group. In our study, however, multivariable analysis showed that the association between the Bengmark protocol and POI was independent of surgical approach. This is in line with the results of Bochner et al. Their randomized trial comparing outcome after ORC and RARC, did not show any differences regarding LOS or complication rates [22]. We acknowledge the limitation of comparing perioperative care between two hospitals. However, many aspects of perioperative care (e.g., postoperative nursing care or perioperative anaesthetic care) are difficult to account for and may confound outcomes even within the same hospital or protocol.

In conclusion, this study showed that early oral nutrition in the ERAS protocol was well tolerated. There were no differences in overall complication rates comparing the two protocols. Importantly, the protocol using nasojejunal feeding was superior considering the frequency of POI. However, because of the retrospective study design, conclusions have to be interpreted with caution. A prospective study is needed to determine if the lower rate of POI in the Bengmark group was due to the use of nasojejunal feeding or other reasons.

## Appendix

See Table 5.

**Table 5:** Overview of pre- intra- and postoperative elements of ERAS and Bengmark protocols

Preoperative care	
Counselling	
ERAS	Patient education about procedure by surgeon at preclinical visit together with specific education about ERAS protocol by nurse practitioner. Written information about ERAS protocol provided
Bengmark	Patient education about procedure and Bengmark tube at preclinical visit, together with written information
Admission	
ERAS	All patients admitted morning of surgery. Consultation by an enterostomal therapist
Bengmark	All patients admitted 1 day before surgery for consultation by an enterostomal therapist and to place a jejunal feeding tube (Bengmark)
Preoperative bowel preparation	
ERAS	None
Bengmark	None
Preoperative carbohydrate loading	
ERAS	Carbohydrate rich drink 2–3 h before surgery for all patients (insulin dependent diabetics excluded)
Bengmark	None
Preoperative fasting	
ERAS	Solid foods up to 6 h before surgery, clear fluids up to 2 h before surgery, then nil oral intake
Bengmark	Solid foods up to 6 h before surgery, clear fluids up to 4 h before surgery, then nil oral intake
Premedications	
ERAS	Acetaminophen 1000 mg on the day of surgery
Bengmark	Temazepam 10 mg the evening before surgery Oxazepam 10 mg and acetaminophen 1000 mg day of surgery SDD: this consisted of the administration of three antibiotics: polymyxin E, tobramycin and amphotericin B. SDD was started the evening before surgery and was given until the first solid oral diet or when enteral feeding exceeded 1 L after surgery
Thromboembolic prophylaxis	
ERAS	Start LMWH prophylactic evening before surgery. Compressive stockings and sleeves for 24 h, starting the morning of surgery
Bengmark	Start LMWH prophylactic evening before surgery. Compressive stockings, starting the morning of surgery
Intraoperative care	
Epidural analgesia	
ERAS	Thoracic epidural (Th11/12) in all patients undergoing ORC, since November 2015 omitted in patients undergoing RARC
Bengmark	Thoracic epidural (Th11/12) in all patients
Antimicrobial prophylaxis	
ERAS	Kefzol 2 g/Flagyl 500 mg started intravenously just before the operation and continued for 24 h
Bengmark	Kefzol 2 g/Flagyl 500 mg started intravenously just before the operation and continued for 24 h
Perioperative fluid management	
ERAS	Restrictive fluid management
Bengmark	Restrictive fluid management
Preventing intraoperative hypothermia	
ERAS	Upper-body air-warming (Bairhugger)
Bengmark	Warming mattress and warming blanket (WarmTouch)
Preventing PONV	
ERAS	Depending on PONV-score calculated at preoperative screening: ondansetron 4 mg at the end of surgery
Bengmark	Depending on PONV-score calculated at preoperative screening: dexamethasone 5 mg and/or droperidol 1.25 mg
Postoperative care	
Nasogastric intubation	
ERAS	Removal after surgery (in recovery, end of day).
Bengmark	Removal 24 h after surgery, unless adhesiolysis, nausea or > 1000 ml production
Drain removal	
ERAS	Removed on POD 2 (if suspect for urinary leakage, creatinine measurement first)
Bengmark	Removed on POD 3 (if suspect for urinary leakage, creatinine measurement first)
Nutrition	
ERAS	POD 0: Start with 1–2 bottles of high calorie nutritional drinks, continue until discharge. Aim for at least 800 ml of oral liquids. POD 1: Light oral diet (bread and liquids). POD 2: Normal oral diet in the absence of nausea, vomiting or abdominal distension
Bengmark	Start with enteral nutrition via Bengmark on POD 0 First 6 h 42 ml/h, second 6 h 65 ml/h, after that depending on dietary need as determined by dietician. Start oral intake depending on peristalsis
Prevention of postoperative ileus	
ERAS	Magnesium oxide twice daily and chewing gum for 5–45 min thrice daily
Bengmark	Magnesium oxide in some patients, depending on bowel movement. Chewing gum as often as possible
Postoperative analgesia	
ERAS	Stop epidural 48 h after surgery. Acetaminophen 1000 mg four times a day starting on POD 0. Diclofenac (50 mg thrice daily) starting before removal of epidural (not in case of impaired renal function)
Bengmark	Stop epidural on POD 4. Acetaminophen 1000 mg four times a day starting on POD 0
Mobilisation	
ERAS	POD 1: 2 h on chair. POD 2: 6 h on chair
Bengmark	Start mobilisation on POD 0, not further specified
Discharge criteria	
ERAS	Normal diet, return of normal bowel function, mobilisation on pre-operative level, able to take care of urinary diversion, adequate oral pain management
Bengmark	Normal diet, return of normal bowel function, mobilisation on pre-operative level, able to take care of urinary diversion, adequate oral pain management

*LMHW* low molecular weight heparine, *ORC* open radical cystectomy, *POD* postoperative day, *RARC* robot-assisted radical cystectomy, *SDD* selective digestive decontamination
